# Supplementary material for: Identification and functional analysis of non-coding regulatory small RNA FenSr3 in Bacillus amyloliquefaciens LPB-18
Source: PeerJ. 2023 May 15;11:e15236. doi: 10.7717/peerj.15236 (PMC10194069; doi:10.7717/peerj.15236)
Supplement: Supplemental Information 4 [file peerj-11-15236-s004.zip › KO/CK-vs-T1_map/map05144.html]

KEGG PATHWAY: Malaria - Reference pathway


|  |  |
| --- | --- |
| **Malaria - Reference pathway** |  |

[
Pathway menu
| Organism menu
| Pathway entry
| Show description
| User data mapping
]

Plasmodium protozoa are parasites that account for malaria infection. Sporozoite forms of the parasite are injected by mosquito bites under the skin and are carried to the liver where they develop into the merozoite form.
Sporozoite invasion of hepatocytes is mediated by parasite surface protein like CSP. Subsequent infection into red blood cells (RBCs) by merozoites causes malaria disease via aberrant cytokine production and sequestration of parasite-infected red blood cells (pRBCs) to host endothelium. Microvasculature sequestration in the brain brings about cerebral malaria that can results in death or persisting neurological impairment. PfEMP1 has been suggested as the key adhesive molecule of pRBCs.

|  |  |  |
| --- | --- | --- |
| Reference pathway | 184% 150% 122% 100% 82% 67% 55% | 图片下载 |
